# Unveiling the Role of Fractionated Graphene Oxide in Nitric Oxide Scavenging

**DOI:** 10.3390/molecules30051069

**Published:** 2025-02-26

**Authors:** Grigoriy R. Chermashentsev, Ivan V. Mikheev, Daria-Mariia V. Ratova, Elena V. Proskurnina, Mikhail A. Proskurnin

**Affiliations:** 1 Department of Chemistry, Lomonosov Moscow State University, Moscow 119234, Russia; chermashentsev96@mail.ru (G.R.C.); darmarrat@gmail.com (D.-M.V.R.); proskurnin@gmail.com (M.A.P.); 2 Research Centre for Medical Genetics, Moscow 115522, Russia; proskurnina@gmail.com; 3 Kurnakov Institute of General and Inorganic Chemistry, Russian Academy of Sciences, Moscow 119071, Russia

**Keywords:** graphene oxide, nitric oxide, chemiluminescence, free radicals, purification, pro-oxidant, antioxidant, kinetic modeling

## Abstract

The feasibility of saturating aqueous anoxic solutions with in situ-generated high-purity nitric oxide (NO) is shown herein. A methemoglobin assay estimated the average nitric oxide concentration to be ca. 20 ± 3 µM. Graphene oxide aqueous dispersions were prepared by ultrasound-assisted extra exfoliation. These dispersions, including unpurified (pristine) samples and samples purified from transition metal impurities (bulk) fractions (bulkGO) and (nano) separated fractions (nanoGO) in a range of 0.5 to 14 kDa were prepared with ppm level concentrations. A robust and reproducible chemiluminescence (CL) assay validated the interaction between graphene oxide and NO in a luminol-based system. The results showed a significant increase in NO scavenging activity within the bulkGO fractions to nanofractions ranging from 14 to 3.5 kDa. The different reaction pathways underlying the transformation of nitric oxide are being evaluated, focusing on understanding how its presence or absence affects these processes. Our kinetic model suggests a significant difference in nitric oxide regulation; nanoGO demonstrates an interception rate seventy-times higher than that achieved through CL quenching.

## 1. Introduction

Graphene oxide (GO) is a graphene-based material that exhibits solubility in neat polar solvents due to the large, oxygenated network of groups on the 2D monolayer of graphite surfaces with sp^2^- (graphene domain), sp^3^-carbon (functional groups domain), and hole-like defects. GO research trends have tremendously risen during the last two decades [1]. The use of GO is promising in numerous branches of science and technology. Here are several trends of their application: (1) in chemistry as a chemoselective surface derivatization reaction on highly reactive sites [2]; (2) in the technology of membranes for water filtration and desalination process due to high proton conductivity [3] and water dispersibility [4]; (3) in biology for electron transfer and hence the bio-oxidation of bacterial cells [5]; (4) in optics, electronics, and optoelectronic applications [6] as a material with a tunable refractive index, absorptivity, and nonlinear optical applications in fluorescent labeling; and lastly (5) in medicine as a material with tuning cytocompatibility for drug delivery [7], biocompatible scavenger-assisted graphene oxide for photodynamic cancer therapy [8], the capacity to modulate the composition of the gut microbiome in adult zebrafish [9], and free radical scavenging capacity [10].

For biomedical purposes, it is crucial to control and manage pure [11], standardized [12], and characterized [13] GO aqueous dispersion samples, which are preliminarily purified by various types of continuous counter current hollow fiber dialysis [14] or conventional ready-to-use dialysis bag (tubing) [15] approaches to remove (a) transition metal impurities from primary oxidants used for graphite exfoliation [16] and (b) oxidative debris (carbonaceous fragments) [17].

GO can be considered as a potential pharmacological drug platform for cancer therapeutics [18] or as an antioxidant agent with free radical scavenging ability with radioprotection [19] in both cell and animal models; it becomes essential to study its effect on the products of cellular metabolism concerning reactive oxygen species (ROS) [20]. Despite the recent debates and trends concerning nanozyme activity [21], there are still reports about the peroxidase-like (nanozyme) activity of graphene [22].

Excess ROS leads to oxidative stress, a pathological condition associated with excessive production of free radicals and their biochemically active intermediates and metabolites, exceeding the protective capabilities of the antioxidant system and leading to destructive consequences and apoptosis. GO is accompanied by undesirable effects for interaction with the cell membrane; the upregulation of NOX1 takes place and induces significant intracellular ROS production [23]. Primary research has focused on the antioxidant scavenging properties of graphene oxide concerning the main primary reactive oxygen species (ROS): superoxide radical anions [24], hydroxyl radicals [25], and hydrogen peroxide [26] (∙O_2_^−^, ∙OH, and H_2_O_2_). But there are no studies on GO interaction with reactive nitrogen species (RNS): nitric oxide (NO) and peroxynitrite (ONOO^−^)—which is a product of reaction with superoxide (∙O_2_^−^). However, related materials like carbon dots have shown high affinity towards NO affected by π-domains and the surface/edge state [27]. Also, the influence of GO on RNS determination has not been confirmed by chemiluminescence assays or other methods.

Numerous studies have established the critical role of the NO molecule for the body as an activator and inhibitor of various metabolic processes. An essential function of nitric oxide is activating the sodium–potassium pump, which leads to the transient hyperpolarization of membranes of vascular smooth muscle tissue [28,29]. The regulatory effect of NO on mitochondrial respiration by inhibiting cytochrome oxidase is known [30]. The presence of soluble NO synthases in brain tissue, especially in the cerebellum and hippocampus, indicates that NO is an important neurotransmitter. It is assumed that nitric oxide is a retrograde messenger of enhanced neurotransmitter formation in presynaptic cells; it leads to prolonged potentiation in the hippocampus and prolonged depression in the cerebellum [31]. This is important for neuronal plasticity, memory performance, and learning [32]. Nitric oxide is also involved in immune system function. Macrophages activated by endotoxins, lipopolysaccharides, or interferon express NO synthase. NO produced by macrophages determines their cytotoxic and cytostatic action on bacterial and tumor cells [33].

Due to high reactivity, nitric oxide is very difficult to prepare and stabilize in aqueous media. To date, there are several approaches which deal with achieving (1) nitric oxide chemical preparation from inorganic components [34] or various organic NO donors, e.g., 1,2,5-oxadiazole 2-oxides (furoxans) [35] and NONOates [36]; (2) nitric oxide electrochemical generation, e.g., from the air [34]; or (3) commercial products of nitric oxide gas cylinders (assay >99%).

The two main methods for measuring NO are electrochemical (for the gas state) and spectroscopic (for aqueous media). They are based on the ability of the NO molecule to take part in radical processes. The problems encountered with this molecule are related to its high reactivity [37] and short half-life, as well as its fast diffusion rate [38] and activity in a wide concentration range from pico- to micro-molar concentrations [39], which makes it necessary to use an appropriate linear response range when employing chemical sensors for NO. Most approaches used deal with the Griess assay after NO oxidation to nitrite [40]. Zinc and various enzymes, e.g., nitroreductase, are added to reduce the formed nitrate [41] with an LOD ca. 1 µM [42]. This technique allows a real-time estimation of NO concentration, which is necessary for medical research. A hemoglobin assay concerning reaction with oxyhemoglobin [43] shows an LOD ca. 1 ÷ 3 µM. The interaction of nitric oxide(II) with oxyhemoglobin proceeds 26-times faster than oxygen so that measurements can be performed without the preliminary removal of dissolved O_2_. The disadvantage of this technique is the need for the initial purification of oxyhemoglobin before use and the absence of redox-active components capable of oxidizing it [44]. Other indirect methods are based on the radiolabeling of L-arginine to determine the NO released by the enzyme group of NO synthases (NOS) through the catalytic conversion of L-arginine to citrulline and NO [45]. The electrochemical approach of measuring NO was measured in situ by a porphyrinic-based microsensor [46].

The chemiluminescence assay has the advantage of high specificity toward NO. Interactions between NO and H_2_O_2_ form peroxynitrite (ONOO^−^), which subsequently reacts with luminol to give the characteristic chemiluminescence. This analysis method requires the absence of oxygen in the atmosphere because in the presence of NO_2_, the signal obtained from nitric oxide is reduced. In contrast to the first approach of detecting NO with O_3_, this one is indifferent to the N$O_{2}^{-}$ and N$O_{3}^{-}$ content with an LOD ca. 100 fM [47]. The chemiluminescence method is excellent for the quantitative determination of NO in biological objects such as arterioles, also widely used in the analysis of human exhaled air, which allows the creation of analytical sensors for monitoring NO as a diagnostic marker of asthma or respiratory infection [48].

A significant advantage of the CL curves is continuous signal registration, which allows us to conduct chemical reaction kinetic modeling, specify their mechanism by computer simulation, and assess antioxidant potential. Previously, modeling of the CL curve shape was successfully applied in the estimation of rate constants of antioxidant reaction of drugs [49] and nanomaterials, e.g., fullerenes C_60_, C_70_, and endofullerenes Gd@C_82_ [50].

This work aims to develop and adapt an analytical model of RNS in situ generation for chemiluminescent assay and the estimation of the pro- and antioxidant properties of GO in this model, which is essential for the purposes of biomedical science and practice. The kinetic modeling performance for identification of the possible mechanism and calculating kinetic parameters of the chemical reactions in the presence of graphene oxide is also important.

## 2. Results

### 2.1. Preparation of Nitric Oxide Saturated Aqueous Solution

**Figure 1:**
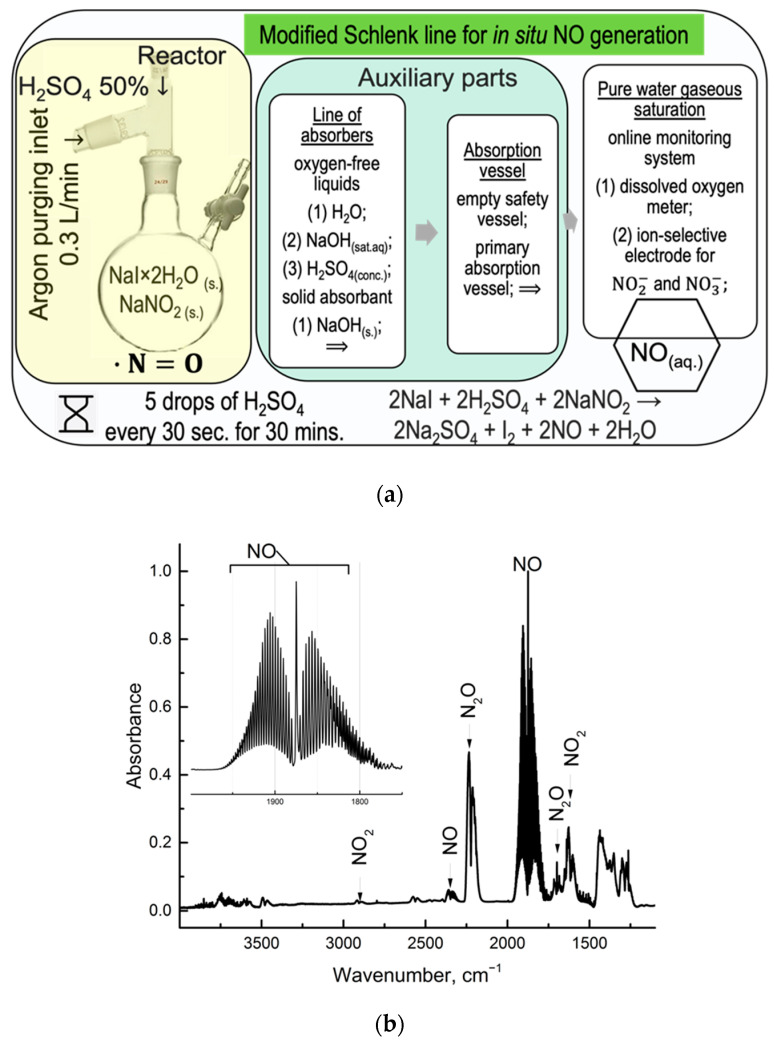
(**a**) A schematic set-up for in situ preparation of nitric oxide saturated aqueous solution with simultaneous byproduct signal registration in the aqueous phase; (**b**) FTIR spectra (gaseous phase) of synthesized nitric oxide after 5 min of starting synthesis.

The procedure for selective chemical generation of nitric oxide has been selected (Procedure 1). It is based on the oxidation of iodide anions by nitrate anions in acidified conditions. We proposed an in situ generation line of NO consisting of the following:

(1) Argon purging line with flow 0.3 L/min;
(2) Schlenk reactor with solid reagents NaI, NaNO_2_, and syringe to drop-by-drop adding 50% H_2_SO_4_;
(3) Axillary parts of set-up including serial connections of absorbers with H_2_O, NaOH saturated solution at 25 °C, H_2_SO_4_ concentrated solution, and an empty safety vessel;
(4) Polypropylene tube filled with solid fine-grained sodium hydroxide ca. 10 g sealed with double-sided absorbent cotton;
(5) Online monitoring system for registering dissolved oxygen, nitrite, and nitrate anion content (see Figure 1a);
(6) Online monitoring requires auxiliary equipment; gas analysis and an exhaust system (for FTIR analysis) are optional additions.

If the yield of the reaction equals 100%, it will correspond to ca. 670 mL (STP) of NO, for the primary reaction is 2KNO_2_ + 2NaI + 2H_2_SO_4_ → I_2_ + 2NO + K_2_SO_4_ + Na_2_SO_4_ + 2H_2_O.

### 2.2. FTIR Analysis of Gaseous Nitric Oxide

FTIR spectra (gaseous phase) of synthesized nitric oxide after 5 min of starting synthesis are presented in Figure 1b. Here, the primary bands of nitric oxide and their derivatives were registered, coinciding with [51] NO_x_ speciation for N_2_O at 2200 cm^−1^, NO at 1850 cm^−1^, and NO_2_ at 1585 cm^−1^. The total area under spectra at the regions 1750 ÷ 1980 was about 25.5%, allowing the NO volume concentration of 4200 ppmv (0.42% vol.) using calibration curves data from [52].

The estimated measurement uncertainty of the results does not exceed 20%. An independent verification (accuracy estimation) of the results was conducted using a gas analyzer directly connected to the gas mixture 3800 ppmv (0.38% vol.). The one-sample Student’s *t*-test passed (*n* = 5, *p* = 0.95).

### 2.3. Nitric Oxide Quantification in an Aqueous Solution

**Figure 2:**
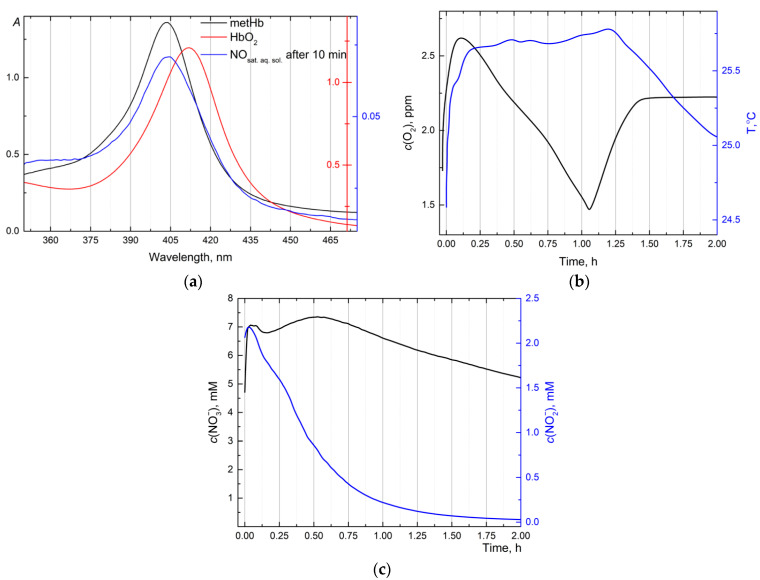
(**a**) Absorbance spectra of native solution of Hb, which consists of an admixture of metHb at 404 nm (solid black line), HbO_2_ solution after prereduction by sodium dithionite (solid red line), *c*(HbO_2_) = 0.85 ± 0.13 mM, and spectra of NO solution after 10 min of saturation with HbO_2_ (solid blue line), *c*(NO) = 3.3 ± 0.3 µM. Collecting signals from probes of dissolved oxygen, nitrate, and nitrite anions after saturation of aqueous solution by nitric oxide during 30 min, (**b**) dissolved oxygen O_2_ (ppm) and temperature (T, °C) and (**c**) nitrate (${NO}_{3}^{-}$) and nitrite (${NO}_{2}^{-}$) anion content (ppm).

The oxyhemoglobin assay has been used to estimate nitric oxide content in an aqueous solution through oxidation to nitrate (Procedure 3) Furthermore, oxygen at storage continuously oxidizes hemoglobin in nonfunctional methemoglobin (metHb). To reduce hemoglobin by prereduction to HbO_2_, sodium dithionite was used as shown by the following schematic chemical equation according to Procedure 3 (Figure 2a).

$$\begin{array}{l} \mathrm{Hemoglobin}\text{–}\mathrm{Fe}(\mathrm{II})/\mathrm{Fe}(\mathrm{III})\;(\mathrm{Hb}/\mathrm{metHb})+{\mathrm{Na}}_{2}S_{2}O_{4}\;\overset{O_{2},H_{2}O}{\to }\\ \mathrm{Hemoglobin}\text{–}\mathrm{Fe(II)}\text{–}O_{2}\;(\mathrm{Hb}O_{2})+{\mathrm{Na}}_{2}SO_{4}; \end{array}$$

An oxyhemoglobin solution was added to the test solution to analyze the NO content, and the degree of conversion to MetHb was determined (Procedure 3) at two wavelengths, 406 and 415 nm [53].

$$\mathrm{Hemoglobin}\text{–}\mathrm{Fe}(\mathrm{II})\text{–}O_{2}\;({\mathrm{HbO}}_{2})+NO_{\left(\mathrm{aq}.\right)}\;\to \;\mathrm{Hemoglobin}\text{–}\mathrm{Fe}(\mathrm{III})+{NO}_{3}^{-};$$

As a result, the nitric oxide concentration after 30 min saturation of ultrapure anoxic water was about *c*_NO_ = 20 ± 3 µM (*n* = 3, *p* = 0.95) (see Figure 2a).

### 2.4. Byproduct Quantification in Aqueous Nitric Oxide Solution

The online signals for dissolved oxygen (Figure 2b) and nitrite/nitrate anions (Figure 2c) as a byproduct were collected during water saturation. Upon argon finally having purged the aqueous solution of nitric oxide, the dissolved oxygen content rapidly rose to 2.6 ppm (ca. 1.5 times) for 5 min according to Henry’s law (Figure 2b). The subsequent gradual drop in oxygen concentration (>0.1 h) was due mainly to the oxidation of nitrite to nitrate.

$${2NO}_{2}^{-}+O_{2}={2NO}_{3}^{-}$$

This behavior is confirmed by Figure 2c, where the nitrite anion content is steadily decreasing, and nitrate anion slightly increases and remains steady. After the complete oxidation of the nitrite anion, it was observed that the oxygen level reached a stationary level, which also correlated with the temperature of the vessel.

### 2.5. Chemiluminescent System: Luminol, Hydrogen Peroxide, and Nitric Oxide—Influences of Byproducts on Chemiluminescence

**Figure 3:**
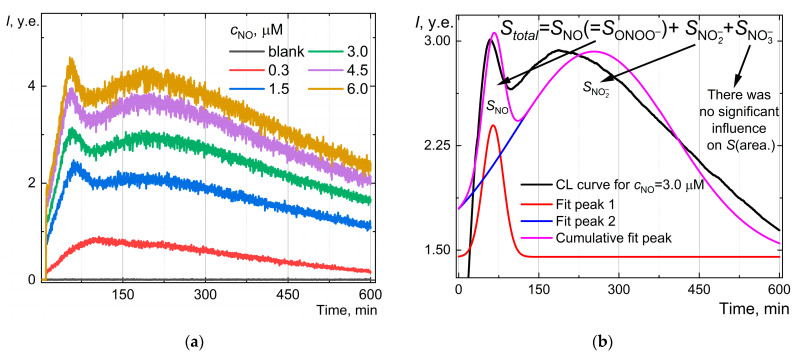

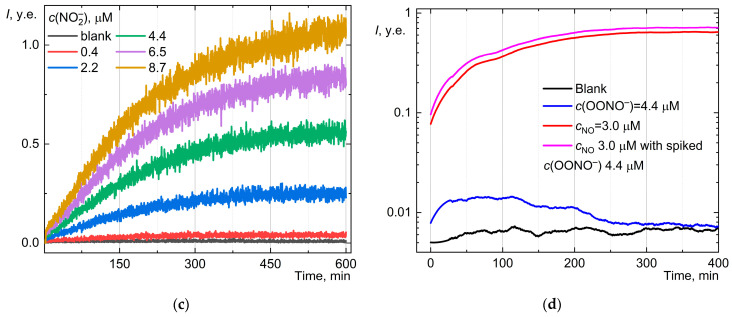
(**a**) Nitric oxide chemiluminescence curves (in a range of 0.3 ÷ 6.0 µM) in the presence of H_2_O_2_ (200 µM) and luminol (2 µM); (**b**) the spectral deconvolution of the chemiluminogram by the Gaussian function for NO (3 µM), H_2_O_2_ (200 µM), and luminol (2 µM)—the solid black line is experimental data, and red, blue, and magenta are simulations; (**c**) nitrite anion (${NO}_{2}^{-}$) chemiluminescence (in a range of (0.4 ÷ 8.7 µM) in the presence of H_2_O_2_ (200 µM) and luminol (2 µM); and (**d**) the influence of a spiked sample of peroxynitrite anions (${ONOO}^{-}$) for nitric oxide chemiluminescence (3.0 µM) in the presence of H_2_O_2_ (200 µM) and luminol (2 µM). The total volume was 1.00 mL; the temperature was 25 °C; all solutions were prepared in PBS, pH 7.4. For cases (**b**,**d**), the chemiluminograms underwent smoothing by the Savitzky–Golay quadratic algorithm with 256 points; (**a**,**c**) present the raw data.

A robust chemiluminescence procedure [48], including luminol (as a probe) and hydrogen peroxide reagents, was selected to determine the nitric oxide content in solution. Also, the influence of a graphene oxide aqueous dispersion regarding nitric oxide CL determination was checked. Figure 3a shows a regular, highly repeatable synthesis-to-synthesis shape of the curve and time of primary maxima. Up to 100 min, a dose-dependent CL was observed; the maximum at 58.3 min corresponds to nitric oxide interactions with luminol and H_2_O_2_. The subsequent recording of the CL curve, up to 10 h, also showed a signal evolution associated with the nitrite anion. The deconvolution of smoothed spectra for a shortened time (up to 400 min) allowed us to confirm and specify two types of Gaussian signals concerning (1) nitric oxide and related peroxynitrite anions (${ONOO}^{-}$) from 10 to 100 min and (2) broad, long-term signals of nitrate anions (${NO}_{2}^{-}$) from 100 min up to 600 min (Figure 3b). The developed CL curve may correspond to the signals from (1) nitric oxide (NO), (2) nitrite ${NO}_{2}^{-}$, (3) peroxynitrite OONO^−^, and (4) nitrate ${NO}_{3}^{-}$. Also, note that the spiked addition of peroxynitrite (Figure 3d) influences the CL signals. The total mathematical area under the curve increases by up to 10–15%. The CL curves for nitrite ${NO}_{2}^{-}$ extremely gradually increase and possess a dose-dependent effect (Figure 3c).

The curve for NO maxima is nonlinear in a range of 0.3 ÷ 6.0 µM. For the linear fit equation *I*_NO_ = (1.54 ± 0.08) × *c*_NO_ (*r* = 0.9910, *n* = 3, *p* = 0.95) (up to 1 µM) of NO detection, the LOQ was estimated as 0.1 µM.

### 2.6. Aqueous Graphene Oxide Aqueous Dispersions: Preparation and Characterization

Having finally conducted a complete chemiluminometry assay with ready-to-operate procedures, we have assessed graphene oxide’s influences on these procedures. The primary graphene oxide samples were off-the-shelf products. All samples (GO powders) were pre-dried [54] under P_2_O_5_ in a desiccator. The total water content was taken into account in further experiments, where $\omega_{H_{2}O}$ = 10.3 ± 1.0% mass. (*n* = 3, *p* = 0.95).

But as we have previously shown, the commercially available GO samples comprise several contaminants like (1) transition metals, (2) oxidative debris, etc. Therefore, we have used highly-purified graphene oxide samples in our work, as described in [15], with extra ultrasonic exfoliation to prepare monolayered graphene oxide aqueous dispersions. Once an aqGO was purified by a dialysis bag, the elemental analysis by ICP–AES and pH measurements were conducted. The quantity of transition metals like Mn, Cr, and Fe did not exceed 0.05 ppm, that of Ti did not exceed 0.25 ppm, and the pH level was 7.8 ± 0.2.

The zeta-potential value of the bulk fraction on average was −35 ± 2 mV, after fractionation, the value having decreased by 30% to −25 mV. The lateral size was ca. 3300 nm. This is the well-known Hammers’ technique of preparing the graphene oxide by oxidation by KMnO_4_ in the presence of H_2_SO_4_ and H_3_PO_4_ [55]. Here, we have adhered to Lerf–Klinowski model of graphene surface functionalization [56], which assumes the availability of sp^2^- and sp^3^-hybridized carbon atoms and different moieties such as hydroxy, carboxy, epoxy, etc.

### 2.7. Raman Spectroscopy Analysis of Prepared Graphene Oxide

**Figure 4:**
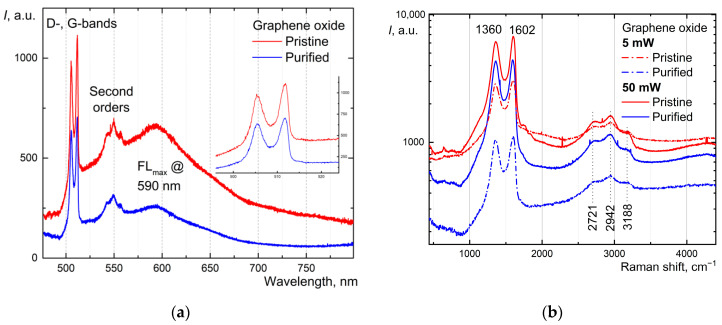
(**a**) Photoluminescence spectra of pristine (solid red line) and purified (solid blue line) GO samples; (**b**) Raman spectra of pristine (red lines) and purified (blue lines) GO samples at laser irradiation powers of 5 mW (all dash-dotted lines) and 50 mW (all solid lines).

Three types of Raman and fluorescence spectra from a solid sample of graphene oxide (GO) and purified graphene oxide were recorded (Figure 4):

1. In the wide range of 477–800 nm (i.e., 177–8640 cm^−1^), to obtain photoluminescence spectra.
2. In the 177–4500 cm^−1^ range (i.e., 477–601 nm), to obtain high-quality Raman spectra of graphite D- and G-bands and their second orders at a probing power of 5 mW.
3. In the range 177–4500 cm^−1^ (i.e., 477–601 nm), to obtain qualitative Raman spectra of graphite D- and G-bands and their second orders at a probing power of 50 mW.

When irradiated, the samples had a small photoluminescence, which gradually burned out to about 40–80% of the original values. Stable spectra are given when the burnout is already accomplished.

Purified and non-purified samples possessed the same ratio of I_D_/I_G_ ~ 1.11, but the total areas under all the spectra reflect in more detail the changes in samples after purification. The total mathematical area ratio for purified/non-purified samples equals ca. 1.5 for spectra registered at 5 mW, but for 50 mW, there are no significant changes.

### 2.8. ATR-FTIR Analysis of Prepared Graphene Oxide and Their Fractions

**Figure 5:**
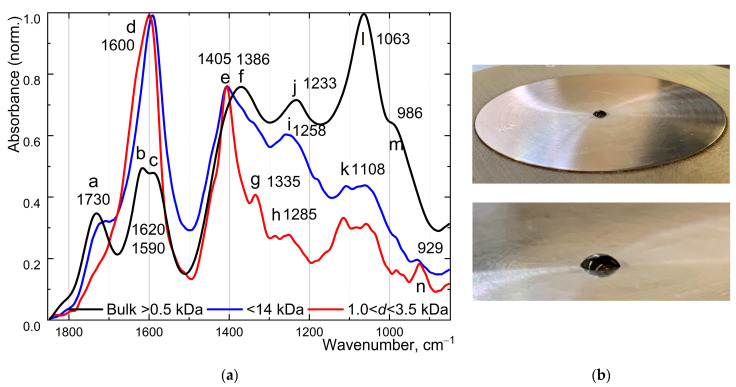
(**a**) ATR-FTIR spectra of purified bulk fraction (by 0.5 kDa membrane) (solid black line) and nanofractions <14 kDa (solid blue line) and 1.0 < fraction < 3.5 kDa GO samples; (**b**) Picture of GO samples (20 µL) deposited on a diamond crystal. The spectra were collected after heating to 50 °C to assist water evaporation.

Analysis of ATR-FTIR spectra (Figure 5a) from GO samples deposited (Figure 5b) as a thin film on a diamond crystal, following heating to 50 °C, enabled their differentiation. All bands for bulk GO samples corresponded to previously reported assignments. The study [57] provides contemporary research concerning the truest and most accurate interpretations of FTIR data of graphene oxide samples.

We observed the changes in oxygen group distribution by FTIR spectra for fractionated samples in comparison with the original sample (Figure 5a). All band assignments are presented in Table 1. Due to the low concentration (sub-ppm) of the fraction between 500 and 1000 Da, FTIR spectra were not collected. Table 1 shows the spectra of two specified fractions (a) 0.5 < *d* <14 kDa and (b) 1.0 < *d* < 3.5 kDa, and their behavior is also described.

### 2.9. Chemiluminescent System Luminol, Hydrogen Peroxide, Nitric Oxide, and Graphene Oxide Aqueous Dispersions

**Figure 6:**
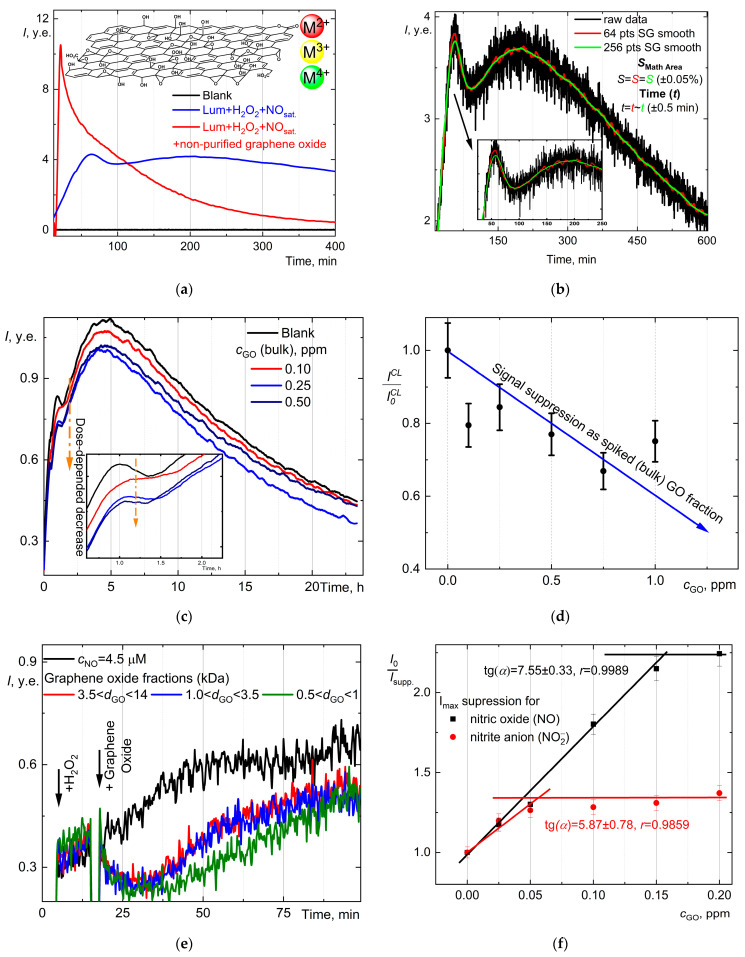
(**a**) Nitric oxide chemiluminescence in the presence of H_2_O_2_ (200 µM) and luminol (2 µM) (solid blue line), the blank experiment (solid black line), and the chemiluminescence curve in the presence of nitric oxide (0.4 µM), H_2_O_2_ (200 µM), luminol (2 µM), and non-purified graphene oxide with transition metal contaminants (M^n+^, where n = 2, 3, or 4) (5 ppm) (solid red line). (**b**) The shape of chemiluminograms: raw data (solid black line), smoothed by the Savitzky–Golay correction algorithm using 64 points (solid red line), and smoothed by the Savitzky–Golay correction algorithm using 256 points (solid green line). (**c**) Chemiluminescence curves for signal suppression in the presence of the isolated bulk fraction >14 kDa. (**d**) Signal suppression of NO chemiluminescence at maximum ~60 min (normalized intensity *I*/*I*_o_, where *I* is intensity in the presence of graphene oxide, *I*_o_ is the intensity of maximum without graphene oxide) based on case (**c**). (**e**) Chemiluminescence curves for signal suppression in the presence of isolated molecular mass-dependent fractions of graphene oxide with size between 14 and 3.5 kDa: $c_{3.5<d_{GO}<14kDa}$ = 110 ± 20 ppb, $c_{1.0<d_{GO}<3.5kDa}$ = 30 ± 5 ppb, $c_{0.5<d_{GO}<1kDa}$ = 10 ± 2 ppb. (**f**) Dose-dependent chemiluminescence signal suppression for a fraction of $0.5<d_{GO}<1kDa$—there are signals of the primary maxima for nitric oxide (at ca. 60 min) and nitrite anions (at ~220 min). The total volume was 1.00 mL; the temperature was 25 °C; all solutions were prepared in PBS pH 7.4. For case (**a**), the chemiluminograms underwent smoothing by the Savitzky–Golay quadratic algorithm with 256 points.

Working with sub-micromolar concentrations assigns limits to the valid signal; the signal-to-noise ratio is deficient. The usage of the Savitzky–Golay algorithm increases visuality and signal usefulness. The 64 = 2^6^ and 256 = 2^8^ points to filter and smooth all data were chosen. The total mathematical area did not change significantly, and the maxima time remained steady (Figure 6c). The 256-point modification obtains a much smoother curve shape without extra random maxima.

The influence of graphene oxide on the luminol-enhanced chemiluminescence of nitric oxide was measured. There were several types of samples.

1. Bulk fraction after synthesis and extra exfoliated by ultrasound: (a) purified (500 Da) sample and (b) non-purified sample (Figure 6a);
2. Fractionated samples of (nano) graphene oxide (nanoGO) with an average mass below 14 kDa (Figure 6b).

For the non-purified samples, a sharp surge in CL intensity was observed due to the inactivation of H_2_O_2_ by transition metal impurities. Also, it is characterized by an inability to interact with nitric oxide and turn on the CL response (Figure 6a). The effects of graphene oxide fractions on the chemiluminescence kinetics of the luminol/H_2_O_2_/NO system are shown (Figure 6b). The CL intensity suppression was observed.

The purified bulk fractions > 14 kDa (Figure 6c) showed nonlinear suppression of CL signals (Figure 6d), the approximation to a half-suppression signal ($c_{½}$) equals ca. 1.5 ppm. For fractionated samples, the concentration level is lower. The halved chemiluminescence intensity (Figure 6e) equals $c_{½}(3.5<d_{GO}<14\;kDa)$ = 75 ± 15 ppb, $c_{½}(1.0<d_{GO}<3.5\;kDa)$ = 20 ± 4 ppb, and $c_{½}\left(0.5<d_{GO}<1.0\;kDa\right)\;$= 10 ± 2 ppb. From the $c_{½}$ values, $3.5<d_{GO}<14\;kDa$ reacts with NO approximately eight-times less than $0.5<d_{GO}<1.0\;kDa$. The tiniest graphene oxide fraction is a more active nitric oxide scavenger. The addition of graphene oxide (small fractions) has no significant effect on the CL signal from the nitrite ion (Figure 6f).

### 2.10. Modeling Chemical Reaction Kinetics

Studying the antioxidant activity of graphene oxide toward nitric oxide through computer simulations begins with a description of a presumable kinetic scheme of reactions. Mathematical modeling of the kinetic profile of CL curves allowed us to estimate the effective rate constants. Also, it helped us to estimate the kinetic antioxidant activity of graphene oxide (dynamic interception ability of radicals). A kinetic scheme of reactions was proposed based on CL signal speciation (Figure 3b).

Here, we present a possible reaction to describe and simulate a stationary level of chemiluminescence signals without oxidants consisting of three processes. The presence of two maxima in the long-term chemiluminescence curves in our experimental data suggests the existence of two independent sources of radicals in our system. The processes involved in the proposed model system are as follows:

General kinetic scheme

1. NO + H_2_O_2_ → ONOO^−^, representing peroxynitrite formation from NO.
2. NO_2_^−^ + H_2_O_2_ → ONOO^−^, representing peroxynitrite formation from NO_2_^−^.
3. NO + H_2_O_2_ → NO_2_^−^ + H_2_O, indicating NO oxidation by H_2_O_2_ to NO_2_^−^.
4. Chemiluminescence process:
  (a) ONOO^−^ + Lum → Lum*, signifying the formation of the excited product.
  (b) Lum* → P + *hν*, representing luminescence.

Additional reactions for aqueous graphene oxide dispersion spike

5. GO + NO → P, scheme representing suppression nitric oxide activity.
6. GO + Lum* → P, signifying chemiluminescence quenching.

The initial simulation conditions are summarized in the Table 2.

**Figure 7:**
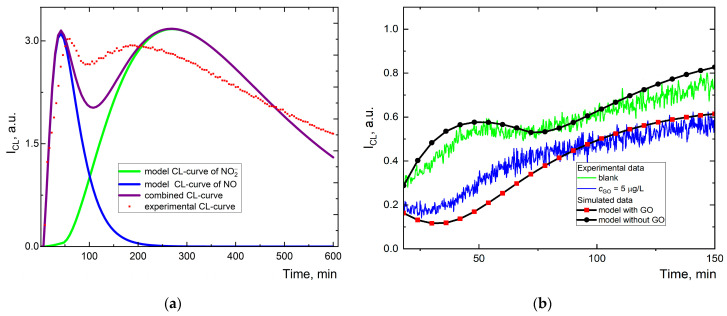
All experiments were performed in 100 mM PBS. (**a**) Long-term experimental chemiluminograms of the luminol (2 mM), H_2_O_2_ (200 μM), and saturated aqueous solution of NO_aq_ (red dotted line), simulated chemiluminescence profile for the NO oxidation process (blue solid line), simulated data for nitrite process (green solid line), and superposition of simulated curves (purple solid line). (**b**) Short-term experimental chemiluminograms of the luminol (2 mM), H_2_O_2_ (200 μM), and saturated aqueous solution of NO_aq_: green is the blank; blue is the GO-containing (small fraction 1 kDa) system; black is the simulated data for blank and GO plots.

Experimental and model plots for chemiluminescence (CL) experiments are shown in Figure 7.

This figure allows for a comparison between the experimental chemiluminescence data and the results of computer simulations for different components of the system, helping us to understand and validate the proposed model. The green solid line (Figure 7) represents the simulated data for the nitrite process, and the purple solid line is a superposition of simulated curves, combining the simulated results for various components of the system. The average deviation (RSD, %) from the experimental data in the initial section (100 min) was 2.2%, which indicates a good agreement between the model data and the experimental results, but moving further the values diverge greatly. In a real experiment, such a strong drop after the first maximum is not observed; the second maximum is reached faster and the glow intensity is observed longer, which indicates a significant influence of side oxidation processes on chemiluminescence.

## 3. Discussion

**Figure 8:**
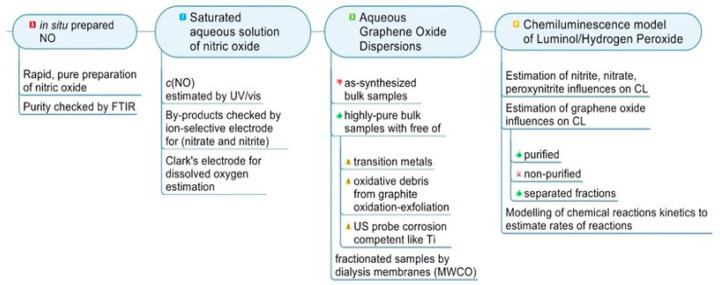
Schematic representations of completed work.

The regulation of oxidative and nitrosative stress by reactions with antioxidants can play a crucial role in preparing new scavenging materials [66]. Due to their unique structure, graphene oxide can serve as a platform for eliminating imbalance between them. Previously, we showed that graphene oxide aqueous dispersions can be obtained in an ultrapure form with scavenging properties possessing superoxide dismutase-like activity [15]. Thus, the results obtained in this research can be summed up along these lines (see Figure 8 below).

1. An improved procedure to saturate aqueous dispersions with control of the oxidized byproduct content was pronounced.
2. Purified aqueous dispersions and their dialysis-separated (nano) fractions can suppress the signals in the nitric oxide CL system.
3. Modeling of chemical reaction kinetics was conducted to estimate the effective rates of reactions.

### 3.1. Preparation of Nitric Oxide and Saturated Aqueous Solution

Nitric oxide is difficult to operate with due to its low solubility, poor storage stability, and high reactivity with air. NO (nitric oxide) does not react with water and hardly dissolves in water [67]. According to [68], the molar fraction solubility for nitric oxide at 25 °C 3.5 × 10^−5^ that coincided with our findings after 30 min of saturation *c*_NO_ = 20 ± 3 µM (see Figure 2a), it is at slightly more than half solubility at this temperature.

The main difficulty in preparing nitric oxide aqueous solutions is the dissolved oxygen content; therefore, we deoxygenate the ultrapure water by boiling before the next steps. In the reaction 2NO + O_2_ ⇄ 2NO_2_, the reactivity of NO_2_ in water precludes direct disproportionation from giving nitric and nitrous acids: 2NO_2_ + H_2_O → HNO_2_ + HNO_3_.

Our proposed approach to the generation of NO is based on the reaction KNO_2_/KI/H_2_SO_4_. However, registering byproducts in the gas state (N_2_O, NO_2_/N_2_O_4_) and in the aqueous state helps to assess the reaction pathway of aqueous solutions of NO saturation. In this work, ion-selective electrodes (ISEs are chosen for rapid and continuous measurements) (${NO}_{2}^{-}$/${NO}_{3}^{-}$), which are as accurate and cost-effective as chromatography (ion-exchange chromatography) [69]. The one advantage of ISEs deals with the possibilities of continuous measuring with rapid response and high selectivity [70]. If the nitrite content (see Figure 2) drops significantly, the nitrate has reached steady-state values, but after two hours of measurement, it starts to fall slightly by 5% every two hours. This is primarily due to (1) the low ionic strength of the saturated NO solution, (2) the need to recalibrate the ion-selective electrodes, and (3) the ion exchange with the internal electrolyte (KCl, 3.5 M), which can significantly influence potential and concentration changes [71] and (4) sustain temperature changes within ±2 °C [72].

As for the determination of the main component (nitric oxide, NO), the system should be an accurate and low-inference without adding extra reagents, e.g., enzymes, etc. The Hb/MetHb system has a better LOD in comparison with other approaches [69].

Finding a stable source of nitric oxide is essential for chemical and biological research. The authors of [73] show that supersaturated nitrite solutions at pH 1.0 can be a suitable container for nitric oxide and its release. Note that when solutions warm from 0 to 25 °C, the excess escapes into the atmosphere. We showed that after storage for seven days of a saturated aqueous solution of nitric oxide at −18 °C, after defrosting, its activity in the CL system fell by 90%. An alternative solution could be the development of nitric oxide chemical containers or a radical trap approach [74]. Such instability behavior after freezing does not allow the development of reference materials for NO, the main recommendations today being in situ generation or saturation with further control concentration.

### 3.2. Characterization of Graphene Oxide

All studies used XRD, Raman spectroscopy, and XPS—established methods for characterizing graphene oxide [2]. Pre-drying materials is a crucial step for the estimation of impurities and the total GO concentration [54]. In a previous work [15], we checked the XPS spectra for the used material. The purification protocol is reproducible, and the used dispersion is highly pure (the total metal content is lower than 1 ppm). As for Raman spectra, it is typical for graphene oxide as described elsewhere [75], although the position of the maximum bands in different publications may differ by 10–20 cm^−1^ in one direction or another. The bands at 1360 and 1602 cm^−1^ are the D- and G-bands of graphite, respectively. The band around 1602 cm^−1^ may also contain the D’-band. The bands at 2721, 2942, and 3188 cm^−1^ are indicated as 2D, D + G, and 2D’ peaks, respectively [76]. They are located on a broad, smooth photoluminescence spectrum of the sample. Increasing the laser power resulted in even more photoluminescence burnout in the samples, making the spectra of the two GO samples almost identical (red and green lines).

Regarding ATR-FTIR spectra, current interpretations and analyses remain ambiguous. Nevertheless, the primary FTIR spectral bands are established. Also, ATR-FTIR spectra for aqueous dispersions are much more appropriate due to the low working quantities of GO (ppm) and volumes (usually no more 25 ÷ 50 mL is isolated). On the contrary, XPS working only with powders needed higher quantities, ca. 100 mg. Using FTIR spectra, we can estimate O-groups species in graphene oxide. As we conclude, the smallest fraction is highly oxidative. Here (Table 1 and Figure 5), epoxies and alcohol groups are absent in the spectra and –COOH appears. This can justify the structural differences between fractions. It should be noted that the group analysis poses a significant and currently unsolved challenge.

This behavior correlated with the electrophoretic size fractionation of GO nanosheets where highly oxidized samples of sizes below 100 nm have the same FTIR spectra. The intensity ratio of most O-containing groups (cm^−1^)—C–O at 1138, C–O–H ~1426, and O–H 3407—increases for the most oxidized sample compared with the least oxidized [77]. Such a behavior was observed for nanodiamonds (crystallite size, 4–5 nm) by DRIFT, where C=O and C–O or C–O–C vibrations were observed at 1770 and 1000–1350 cm^−1^, respectively, for highly oxidized samples [78,79]. Additionally, the paper [80] reports that MAS NMR data indicated comparable chemical structures for the fragments (50–2 kDa), yet smaller graphene demonstrated superior photoemissive properties. Previously, we have shown that the thermal diffusivity of the fraction (all studied in this work) decreased for nanographene in comparison to water [81].

Speaking of nanographene [82] fractions, firstly, it is important to highlight that the water peak is an indicator of water presence within the graphene oxide (GO) sample, either as intercalated (interplane) or crystallized water. Namely, (a) for the bulk graphene oxide fraction, at 1620 cm^−1^, a distinct and reproducible water signal is observed after drying the sample and maintaining it for 5 min on the crystal. (b) Smaller graphene oxide fractions (nano GO) showed no interplanar water and no peak at 1620 cm^−1^. It is likely that these smaller fractions lack a well-ordered layered structure, allowing water to evaporate upon heating on the crystal, resulting in shifting to a clear band at 1600–1590 cm^−1^.

Secondly, for particles with a mass of 1 kDa, their size can be estimated using the density of graphite oxide [83] (1.36 g/cm³), yielding a diameter of ca. 6.2 nm (see Appendix A). Assuming sphericity and excluding the hydrodynamic shell, such particles are challenging to measure directly due to rapid aggregation into larger clusters. Therefore, it is more practical to categorize them by mass fractions (in kDa) [80] rather than particle size.

### 3.3. Chemiluminescence Analysis Systems

From the data in Figure 3, we obtained that nitrate ions (SRM used) do not exhibit chemiluminescent properties in the Lum-H_2_O_2_ system. Nitrate ions do not contribute to the chemiluminescence of saturated aqueous solutions of NO, which is consistent with [47]. In [84], under the UV-C region (222 nm), ${NO}_{2}^{-}$/${NO}_{3}^{-}$ underwent photochemical conversion to peroxynitrite (ONOO^−^) in which the connection ${NO}_{2}^{-}$/${NO}_{3}^{-}$ was separated by IC. Due to our standard conditions, only ${NO}_{2}^{-}$ can be converted long-term to ONOO^−^ by H_2_O_2_ oxidation.

We are convinced that peroxynitrite interacting with the probe causes CL. This is shown in most works with a novel synthesized probe [85] and well-known luminol as a probe [86]. For our conditions, we prove this by the synthesis of peroxynitrite, where the time of occurrence of CL from saturated NO solution coincides with the time of CL from peroxynitrite. NO also reacts rapidly with superoxide (O_2_^−^) to form peroxynitrite (ONOO^−^) [87], and the superoxide can form in our conditions. But ${NO}_{3}^{-}$ does not affect the CL, since we have observed the following forms in solution: NO (transformed to ONOO^−^), and ${NO}_{2}^{-}$ long transformed to ONOO^−^. Also, in the maxima position of peroxynitrite (Figure 3d), there is a long kinetic release up to 300 min at the steady highest position after the first 100 min, which may correspond to the rapid conversion of nitric oxide into peroxynitrite in H_2_O_2_/Lum media.

All subsequent NO system analyses that incorporate graphene oxide necessitate the purification of GO samples for several reasons.

1. Metal impurities [88] can influence reagents:
  - Mn^n+^ can decompose H_2_O_2_ (Figure 6a);
  - Fe^2+^ could be involved in a Fenton-type process with NO [89];
2. Carbonaceous highly oxidized fragments (debris) can act as follows:
  - Quench CL or photoluminescence [90];
  - Induce “imaginary” fluorescence not from graphene oxide [91].

We should realize two types of GO samples to explain the CL dependences obtained in this study. The first one is a bulk fraction where all sizes of graphene oxide exist that consist of a distribution of fractions. It can be a µm sized fraction prepared by vacuum filtration [92] and nano-sized fraction prepared by dialysis protocol [80]. The nanofraction is deposited on the micron-fraction by various interaction types and non-covalent bonding [93]. The more oxidized fraction of graphene oxide (low kDa) has a greater number of groups on the surface [94], a smaller number of layers [95], greater surface accessibility, other things being equal, and possible chirality [96]. We have observed that the smallest fractions (1–3.5 kDa) better suppressed the CL of NO. We can suggest a fraction for nitric oxide scavenging activity that transforms to the peroxynitrite anion as GO (bulk) < GO (3.5 ÷ 14 kDa) < GO (1.0 ÷ 3.5 kDa) < GO (0.5 ÷ 1.0 kDa). Using the average molecular weight cut-off for each bag, we can rely up the molar concentration showing half-suppression of the CL signal, but this estimation does not embrace the active site quantity [97,98]. The $c_{½}$(nM) GO (3.5 ÷ 14 kDa), 13.3 < GO (1.0 ÷ 3.5 kDa), 8.8 < GO (0.5 ÷ 1.0 kDa), 8.6. Usually the quantity of any O-groups around µmol/g makes it possible to conclude that the “truly” active concentration of graphene oxide lies in the range of pM to fM.

This behavior initially appears linked to an increased electron affinity (EA) value; however, it shows that both EA and electrical conductivity increase when the graphene oxide sample is reduced [99] (reduction in the quantity O-containing groups). Speaking of electron affinity, it determined the reactivity of fullerenes regarding peroxyl radicals [50]. Thus, functional oxygen groups play a key role in the reactivity of graphene oxide [20] regarding radicals (e.g., NO) in the fine fraction.

### 3.4. Modeling and Estimation of Effective Rate Constants

One unresolved issue remains in the molar concentration of graphene oxide, or more precisely, molar concentration of species and active groups. This issue continues the trend, not only for graphene chemistry but also previously for nanodiamond surface reactivity [100]. Nowadays, it is impossible to determine due to the changeable molecular C_x_H_y_O_z_ ratio [2], sample aging [101], and variation in fraction size composition [77,93]. There have been many attempts to estimate chemical formulae according to CHNSO analysis [54] and using van Krevelen diagrams [102], but this ratio does not have any chemical species. Also, there is no evidence concerning which oxygen groups work as the active (or even reactive) sites of the graphene oxide plane and take part in the suggested reaction mechanism. To date, only total mass concentrations by electrochemical methods [103], spectroscopic methods [104], or the molar concentration of –COOH have been determined [105]. But the lack of molar concentration is a big issue for estimations of the reaction rate constants.

To simulate the kinetics profile of chemical reactions and clarify the mechanism of action in the CL system in the presence of luminol/H_2_O_2_/nitric oxide and influence graphene oxide on CL, we note the proposed kinetic scheme of reactions (above). The general important suggestion deals with the two-way formation of peroxynitrite ions by (1) NO + H_2_O_2_ = ONOO^−^; (2) NO + O_2_ = NO_2_^−^; NO_2_^−^ + H_2_O_2_ = ONOO^−^. After this, processes associated with peroxynitrite ions occurred by (1) ONOO^−^ + (Lum)LH^−^ = NO_2_·+ L^−^ + OH^−^; ONOO^−^ + (Lum)LH^−^ = NO_2_^−^ + OH^−^ + L (where L is luminol).

In the proposed reaction scheme, two pathways from NO and NO_2_^−^ form peroxynitrite; however, if two chemical processes share a common source (NO) and produce the same product (ONOO^−^), then the kinetics will always show one maximum on chemiluminograms.

Three primary radicals are simultaneously present in the vascular bed: hydrogen peroxide, superoxide anion radicals, and nitrogen monoxide. When NO interacts with hydrogen peroxide and superoxide, peroxynitrite is formed, so oxidative and nitrosative metabolism are closely related. Increased levels of hydrogen peroxide or superoxide (oxidative stress) always lead to disturbances in NO metabolism and are associated with nitrosative stress. Researchers often use a complex model of oxidative/nitrosative stress based on hydrogen peroxide and a NO donor for model experiments on stress and antioxidants [106].

**Figure 9:**
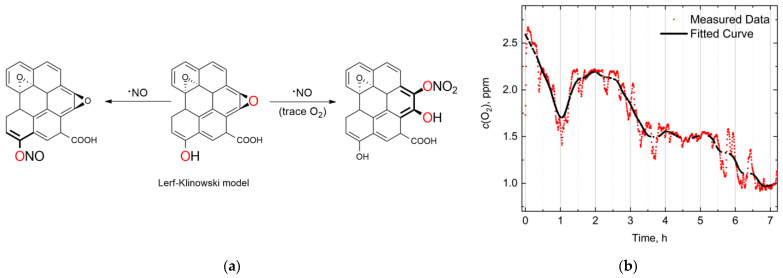
(**a**) Proposed binding sites of nitric oxide on graphene oxide: interactions with phenolic (left) and epoxide (right) groups. (**b**) Experimental measurement of O_2_ content in nitric oxide-saturated solution using a Clark-type electrode.

It is challenging to say exactly which group is responsible for the reactivity of graphene oxide regarding NO; we assume it could be phenols or epoxides. The quantity of epoxy groups are in the µM range (up to 1.5) [107], which could be estimated by XPS after drying the samples. Nitric oxide can be potentially bound to the phenolic [108] and epoxy [109] parts of graphene oxide because of their high reactivity [110]. Figure 9a showed the reaction pathway of nitric oxide with GO under the model of Lerf–Klinowski [56]. The ring-opening reaction of epoxy occurs in the presence of an oxygen trace quantity. Figure 9b shows that O_2_ gradually decreased in 1 h, which correlates with the CL signal in neat NO solution (Figure 3a) and in the presence of GO (Figure 6e). The 100 min process can lead to byproduct interactions that potentially generate CL signals.

Since strengthening one process will lead to weakening the other and vice versa, two maxima may appear on the CL curve only if two independent chemical processes have different sources of radical. Signal letdown (Figure 3a from 50 to 150 min) is possible only if the first source of radicals ends and the second source has not yet ended. However, if the quantity of nitric oxide ends here, then NO_2_^−^ can no longer be formed. It looks like a non-competitive inhibition in fermentation kinetics. In conclusion, there are two independent processes here, with two separate sources of radicals.

After conducting experimental estimations of the model system (NO + Lum + H_2_O_2_), we examined the reactivity of graphene oxide towards nitric oxide. Considering these findings, we propose incorporating two additional reactions into the kinetic scheme of reactions, which include the following:

1. Nitric oxide suppression by binding or transformation by graphene oxide oxygen groups [56], which can act as a potential antioxidant.
2. Chemiluminescence quenching by a conjugate net of graphene oxide [111] or “true” graphene sites [112], or even oxidative debris [17] due to the fact we cannot be certain of the absolute purity of graphene oxide preparations after dialysis purification.

These reactions are significant because graphene oxide has antioxidant properties [113] and can quench chemiluminescence [114,115]. The curve’s behavior in Figure 3b up to 100 min shows it is an independent radical source. However, after 24 h of incubation in the zebrafish gastrointestinal tract, graphene oxide degrades due to nitric oxide [36], and inhibiting this degradation leads to increased inflammation, characterized by heightened neutrophil infiltration. This aspect was beyond the scope of our study.

The experimental and calculated plots exhibit a substantial similarity, with RSD being no more than 3% for calculations with and without GO. The simulation shows that the nitric oxide interception by GO is consistently 70-times higher than the quenching CL (Table 2). The complex structure of GO prevented us from calculating concentration-dependent antioxidant plots. To the best of our knowledge, this is the first information regarding the reaction rates of nitric oxide with graphene oxide. We can compare this most closely with the reaction rates of NO with Hb [116], although they are not equal. The existing literature lacks reaction rates with graphene oxide. Because NO is a reactive radical, it accurately describes biomolecules in processes where reactions with NO occur much faster.

### 3.5. Comparative Analysis of Graphene Oxide as a NO Scavenger: Performance and Benchmarking

A comparison of the NO-scavenging performance of graphene oxide with other compounds obviously requires a separate pool of experiments conducted for the developed system under the same conditions. Another important point is that we do not yet know which compound NO is converted into as a result of the reaction with GO. We can hypothesize that these are nitrates or peroxynitrite, but finding this out also requires a separate study. Therefore, we cannot yet discuss graphene oxide in the context of nitrosative stress. However, we can compare the performance of GO in terms of scavenging activity with other data from the literature. In the body, the NO balance is regulated, in particular, by its reversible capture by proteins, such as myoglobin [117], hemoglobin [118,119], and cytochrome c [120,121], but not antioxidant enzymes. Comparing the effectiveness of superoxide dismutase, catalase, 2,2,6,6-tetramethyl-1-piperidinoxyl (TEMPO), *N*-acetylcysteine, dimethylthiourea, and uric acid, the authors concluded that only TEMPO was an excellent NO antioxidant, which effectively reduced the suppression of p38 MAPK and p53 stress cascade signaling, NO production, peroxynitrite, and the nitration of protein tyrosine residues [106]. Among natural antioxidants, hydroxystilbenes (oxyresveratrol and resveratrol) have the ability to scavenge NO and also act as inhibitors of iNOS expression [122]. In these experiments, the authors used NO solutions with a concentration of 7.7 μM. Since NO participates in electrochemical reactions, sensors are being developed to quantify it, e.g., based on heat-denatured cytochrome *c* and a radical scavenger, 2-(4-carboxyphenyl)-4,4,5,5-tetramethylimidazoline-1-oxyl-3-oxide (C-PTIO) [123]. The dynamic ranges of these sensing systems were 0.5–4 μM and 0.05–100 μM, respectively. PTIO can be regarded as a “standard” NO scavenger against which to judge the effectiveness of other scavengers, such as uric acid, caffeic acid, and Trolox, which have also proven to be very effective NO scavengers [124]. Comparing the dynamic range of our graphene oxide-based system to 0.3–10 μM, we can state that its scavenging performance is comparable to that of PTIO.

## 4. Materials and Methods

### 4.1. Reagents

Buffer solution and reagents for purification of graphene oxide: All chemicals were chemically pure assay-grade reagents. Phosphate-buffered solution (PBS) with a concentration of 100 mM and pH 7.4 was prepared by dissolving a sample of 13.6 g KH_2_PO_4_ (Sigma-Aldrich, St. Louis, MO, USA) in 1.00 L of ultrapure water. The required pH value of 7.4 was adjusted using granulated KOH (Sigma-Aldrich, St. Louis, MO, USA) and concentrated HCl solution (PanReac, Barcelona, Spain), controlling acidity, if any, with a Mettler Toledo pH meter (MettlerToledo, Greifensee, Switzerland). The pH meter was calibrated daily before analysis according to the recommendations in ISO 23497:2019 [125]. Ethylenediaminetetraacetic acid (EDTA) and sodium carbonate (Na_2_CO_3_) (chemically pure grade) (LLC RusChem, Moscow, Russia) were used to purify graphene oxide dispersions and precondition the dialysis bags.

Chemiluminescent reagents: Working hydrogen peroxide solutions were prepared by diluting 30 *w*/*v*% of H_2_O_2_ solution (Sigma-Aldrich, St. Louis, MO, USA) with deionized water, preliminarily checked as determined by permanganometry assay. Luminol dry substance 97% (HPLC-grade assay) (Sigma-Aldrich, St. Louis, MO, USA) was used as a chemiluminescent probe (CL-probe). Potassium permanganate aqueous solution *c*(KMnO_4_) = 0.05 M was standardized against di-sodium oxalate (CRM) (LLC Khimsnab-SPB, Saint Petersburg, Russia).

Nitric oxide synthesis: Solid NaNO_2_, NaI × 2H_2_O, and concentrated H_2_SO_4_ (LLC RusChem, Moscow, Russia) were used for nitric oxide synthesis in a Schlenk flask. Gaseous argon (99.993% assay) was used from LLL AirLiquid, Moscow, Russia.

Nitric oxide determination: Hemoglobin human (Hb) lyophilized powder (Sigma-Aldrich, St. Louis, MO, USA) and sodium dithionite (Na_2_S_2_O_4_) (LLC RusChem, Moscow, Russia) were used.

**⚠**Caution. All manipulations with nitric oxide ought to be conducted in fume hoods due to the rapid oxidation-conversion to nitrogen dioxide. Exposure to this could lead to pulmonary edema.

### 4.2. Materials

Graphene oxide (GO) (LLC Rusgraphene, Moscow, Russia) was used. Prior to manipulation, commercial samples of graphene oxide were put into desiccators with P_2_O_5_ (LLC RusChem, Moscow, Russia) for at least one week to remove inter-layered water [126].

### 4.3. Materials Purification

Graphene oxide and purified graphene oxide aqueous dispersions have been prepared as described elsewhere in [15] using dialysis with a 0.5 kDa membrane to remove inorganic admixtures and oxidation debris.

Also, the graphene oxide (nano) fractions have been isolated as follows:

(1) Bulk fraction >14 kDa;

(2) Small fraction with lateral size between 14 and 3.5 kDa;

(3) Tiniest fraction from 3.5 to 0.5 kDa.

Cellulose dialysis bags with pore sizes of 14, 3.5, and 0.5 kDa with a width of 25 mm were used from LINYEYUE Laboratory Supplies Factory Store (Shijiazhuang Linyue Trade Co., Ltd., Shijiazhuang, China).

All dialysis bags were preconditioned to remove inorganic and organic contaminants and stabilizers for 48 h using 200 mL in total of an EDTA (25 µM), H_2_O_2_ (5 µM), and Na_2_CO_3_ (0.75 µM) mixture solution, which was stirred in the first 24 h, and then followed by a rinse with deionized water. Ready-to-use membranes were stored at 4 °C in deionized water for no longer than one month. All dialysis experiments (preconditioning and purifications) were conducted stirring at 0.4 krpm in a dark place.

### 4.4. Equipment for the Spectroscopy Characterization and Analysis

Raman spectroscopy: A Horiba Jobin Yvon LabRAM (Jobin Yvon, Oberursel, Germany) HR-800 laser confocal Raman spectrometer was used. The excitation source was a diode-pumped solid-state laser (at a wavelength of 473 nm, and a power adjustment range at 5 or 50 mW). The radiation was focused into the region of 1 ÷ 2 µm. The system for recording the reflection spectra consisted of a monochromator with a 600 lines/mm grating and a CCD camera. The mode of spectra registration was an average of 3–5 spectra by 2–10 min of accumulation in one window. The spectra were normalized to instrument sensitivity. A piece of graphene oxide film was placed on a silicon substrate, and then the reflection spectrum was recorded.

FTIR spectroscopy: FTIR measurements in the near IR region from 700 to 6000 cm^−1^ were performed using a Vertex 70 (Bruker, Hamburg, Germany) with a liquid nitrogen-cooled MCT detector [127]. FTIR measurements of the gas mixture were carried out in a gas cell, including the glass body, o-rings, and cell holder, with a 50 mm path length and a diameter of 25 mm (Pike Technologies Inc., Madison, WI, USA). The spectra registration conditions are the number of background scans, 64; number of sample scans, 64; and resolution, 0.5 cm^−1^. The background spectrum was recorded relative to an inert atmosphere (argon). The spectrometer was constantly purged with dry nitrogen. As for aqueous samples, the collecting conditions are in good accordance with [81].

Chemiluminometry: Chemiluminescence was recorded in 2.0 mL Röhren tubes on a 12-channel Lum-1200 chemiluminometer with an original software product PowerGraph (version 3.3) (DISoft, Moscow, Russia). Weighing of all reagents was carried out on the analytical scales “Voyager” (OHAUS Europe, Nanikon, Switzerland). All aliquots were taken using a set of Proline^®^Plus Mechanical Pipette (Sartorius, Helsinki, Finland) with variable volumes of 10–100, 20–200, and 100–1000 µL. Tolerances for pipettes in the laboratory have been checked according to the ISO 8655-2:2022 [128] standard for pipette testing every 3 months. Tubes with solutions were stirred on a Vortex V-3 “ELMI V-3” shaker (ELMI, Riga, Latvia) at 1 krpm for 20 sec before analysis. For all cases, two replicates of chemiluminograms were recorded, and the signal’s relative standard deviation (RSD in %) for all points has not exceeded 5.

Analytical signals in chemiluminometry: The raw data of processed signals were used, unless otherwise agreed. In some cases, the Savitzky–Golay filter for the 64- or 256-point algorithm was applied to visualize signals and smooth data. *c*_½_ is the concentration of half intensity diminishing (50%) of the initial CL value. Also, the total mathematical area S under the CL curve has been used as an analytical signal in nitrogen speciation analysis: (1) nitric oxide (NO); (2) nitrite${NO}_{2}^{-}$; (3) peroxynitrite OONO^−^; and (4) nitrate ${NO}_{3}^{-}$ (Figure 3).

Other instruments: A magnetic stirrer with a hot plate (ISO Lab, Isolab Laborgeräte GmbH, Eschau, Germany) and a magnetic stirrer MS3 JoanLab (Joanlab Equipment Co., Ltd., Huzhou, China) were used. An Agilent Cary 4000 spectrophotometer (Agilent, Mulgrave, Australia) was used for UV/Vis spectra recording for nitric oxide determination and species of Hb in aqueous media. A 10 mm quartz cuvette was used. The scanning rate was 60 nm/min, and the data interval was 1 nm in all cases. An inductively coupled plasma–atomic emission spectrometry (ICP-AES) Agilent 720 (Agilent, Mulgrave, Australia) was used. An ISC-1600 ion chromatograph (Thermo Fisher Scientific, Oxford, UK) equipped with a Dionex IonPac chromatographic column (Thermo Fisher Scientific, Sunnyvale, CA, USA) AS4 (4 mm I.D., length 250 mm) with a Dionex IonPac AG4 precolumn (4 mm I.D., length 50 mm) was used for accuracy estimation for the anion analysis of NO_2_^−^ and NO_3_^−^. An Expert-001-2 O_2_-m (Econix-Expert, Ltd., Moscow, Russia) equipped with a Clark-type electrode, ${NO}_{2}^{-}$ and ${NO}_{3}^{-}$ ion-selective working electrodes, and an Ag|AgCl (E = +222 mV at 25 °C) reference electrode (Expert, Ltd., Moscow, Russia) was used. Signals were collected using MultiTest software (version 1.0; Expert, Ltd., Moscow, Russia). SRM materials (standards): (a) 1000 ppm anion-multi-element solutions were received from Merck, (b) 100 ppm 21 Element ICP Calibration/Quality Control Standard were obtained from Inorganic Ventures (Christiansburg, VA, USA). To determine the NO_x_ (NO/NO_2_) content in the gas mixture, we used the portable gas analyzer OPTIMA 7 Biogas Analyzer (MRU, Hamburg, Germany). The zeta-potential and particle size of GO were measured using a Malvern Instruments Ltd. (Malvern, Worcestershire, UK) Zetasizer Nano ZS.

Data treatment: All calibration parameters were estimated according to the IUPAC recommendations for presenting the results of chemical analysis published elsewhere in [129]. The measurement results are presented as mean values with confidence intervals (*p* < 0.05 or *p* = 0.95) under the requirements for the competence of testing and calibration laboratories ISO/IEC 17025:2020 [130]. All statistical analysis and figure illustrations were performed in Origin 2017 SR1 b9.2.257 software (version 92E) (OriginLab Corporation, Northampton, MA, USA).

### 4.5. Procedures

Procedure 1. In situ chemical generation of nitric oxide

Distilled water was boiled for 10 min and kept in an ultrasonic bath for 15 min to remove oxygen. The absorbers were filled with distilled water, saturated NaOH solution, and concentrated H_2_SO_4_. A solid NaNO_2_ mixture (8.106 g, 0.11 mol) and NaI×2H_2_O (5.57 g, 0.03 mol) were placed into a Schlenk’s vessel. A 50% H_2_SO_4_ aqueous solution was poured into an additional funnel, and the set-up was purged with argon (Ar). Nitric oxide was saturated with 100 mL of distilled water in a single-necked flask, adding acid to the reaction mixture dropwise at a rate of 5 drops per 30 s for 30 min. During saturation, oxygen, nitrite, and nitrate values were processed. FTIR spectra of the gaseous mixture were recorded after 5 min of in situ generation under laminar NO(Ar) gaseous mixture passing through.

Procedure 2. Preparation of peroxynitrite ion solution in aqueous media

A total of 3.0 mL of H_2_O_2_ (35 wt.%, *d* = 1.07 g/mL) was added to a 50.0 mL volumetric flask to prepare a solution of hydrogen peroxide (0.70 M) acidified with hydrochloric acid (0.60 M), then 2.4 mL of HCl (38 wt.%, *d* = 1.19 g/mL) was made up to the mark with deionized water (hereinafter, solution I). A total of 2.07 g of sodium nitrite was added to another 50.0 mL volumetric flask to prepare (0.60 M) NaNO_2_, dissolved in ~25 mL of deionized water, and then made up to the mark (hereinafter, solution II).

In a 50.0 mL polypropylene tube, 10 mL of solution I and II were mixed. An equal volume of sodium hydroxide solution (1.5 M, 6.0 wt%) was immediately added to the resulting solution, and a light-yellow solution formed. To remove excess H_2_O_2_, ~30 mg of MnO_2_ was added to the reaction vessel. After 15 min, the solution was filtered through a 0.45 µm cellulose syringe membrane filter. Then, the ($c_{{ONOO}^{-}}$, M) content was determined spectrophotometrically at 302 nm with apparent molar absorptivity *ε*_302 nm_ = 1670 M^−1^ × cm^−1^ [131]. To reuse the solution, the prepared sodium peroxynitrite was frozen at −18 °C, after warming to room temperature, peroxynitrite was determined spectrophotometrically [132].

Procedure 3. Oxyhemoglobin assay for nitric oxide determination

A total of 25.0 mg of hemoglobin was dissolved in 1.00 mL of phosphate-buffered solution, then 5.0 mg of sodium dithionite was added to the solution and left in air for ca. 10 min in a sealed 15 mL polypropylene tube. The color of the solution was observed to change from brown to dark red. The concentration and purity of HbO_2_ in the resulting solution were established spectrophotometrically at ε_415 nm_ = 1.31 × 10^5^ M^−1^ × cm^−1^ by HbO_2_ absorbance. To determine NO after 30 min of saturation (according to Procedure 1), 0.5 mL of 1 mM oxyhemoglobin solution was added to 75.0 mL of aqueous nitric oxide saturated solution. The absorption spectrum was recorded, and the degree of HbO_2_ conversion to MetHb at 406 nm was estimated [53].

Procedure 4. Luminol chemiluminescence probe in the presence of H_2_O_2_ for the analysis of nitric oxide

Nitric oxide in vitro activity in the presence of GO samples was studied using the chemiluminescence probe luminol and the H_2_O_2_ system. A solution of luminol 2 mM was prepared by dissolving a sample in PBS with pH 7.4. Hydrogen peroxide 0.2 mM solution was prepared by diluting the 37 *w*/*v*% H_2_O_2_ stock solution with ultrapure water. Background signals of luminescence were recorded for 4 min. An aliquot of H_2_O_2_ (50 µL, 0.2 mM) was added, then, at 18 min, an aliquot of the saturated aqueous solution of nitric oxide (150 µL, 20 µM), any type of graphene oxide aqueous dispersion (50 µL, ca. 1 g/L), and PBS up to a 1.00 mL total volume was added. All samples were analyzed in triplicate at 37 °C, and signal registration occurred up to 720 min. As the analytical signal, the total mathematical area for a specific time interval (600 min) under the CL curve for the test and blank experiments (without GO) was used.

Procedure 5. Fractionation of graphene oxide aqueous dispersions (preparation (nano) graphene oxide)

The bulk solution of graphene oxide (25 mL) was placed into a dialysis bag (14 kDa), and 800 mL of deionized water was added. The dialysis was conducted for 7 days. Having finished the dialysis, dialysate with a small fraction (3.5 < fraction < 14 kDa) was concentrated up to ca. 25 mL using a rotary evaporator for 4 h.

The small fraction (3.5 < fraction < 14 kDa) of graphene oxide (25 mL) was placed into a dialysis bag (3.5 kDa), and 800 mL of deionized water was added. The dialysis was conducted for 7 days. After finishing the dialysis, dialysate with the tiniest fraction (3.5 < fraction < 0.5 kDa) was concentrated up to ca. 25 mL using a rotary evaporator for 4 h.

## 5. Conclusions

Chemiluminescence and chemiluminometry (CL) methods are successfully used to analyze radicals and estimate the potential of nanomaterials for their scavenging. This method has proven itself in the analysis of the radical scavenging activity of fullerenes and their derivatives. An important feature of CL is the ability to assess the antioxidant potential of nanomaterials, which includes antioxidant activity (effective reaction rate constants) and antioxidant capacity (the total number of absorbed radicals).

This study uniquely combines CL analysis of nitric oxide with graphene oxide and its fractions. This approach was developed for bulk unpurified, purified, and fractionated samples. The (nano) fraction gives the best efficiency in radical scavenging.

We assume that this work will allow the development of a fully validated approach in the future to determining NO in the presence of graphene oxide. It will also facilitate the development of standardized nanomaterial samples for assessing radical scavenging potential.

## Figures and Tables

**Table 1:** FTIR spectra analysis of the bulk and fractionated samples.

Wavenumber, cm^−1^	Band Assignment in a Graph	Group in a Bulk GO Fraction Purified by 0.5 kDa Membrane	Fraction < 14 kDa	1.0 < Fraction < 3.5 kDa	References
1730	a	C=O stretching	Lowered	Disappeared	[57]
1620	b	H_2_O bands	Disappeared (overlapped?)	Disappeared (overlapped?)	[58]
1590	c	C=C (aromatic)	Increased	Shifted to 1600	[59]
1600	d	C=C or highly conjugated, hydrogen-bonded carbonyl	shifted to 1590	Appeared	[60]
1404	e	−C(=O)−O	Noticeable increase	Noticeable increase	[61]
1368	f	–C–O–H bending	Significantly lowered	Disappeared	[57]
1335	g	Unclear	Unclear	Appeared C–N or COOH	[62]
1285	h	O=C–OH	Did not appear	Appeared	[61]
1258	i	Water protonates the carboxylate groups giving rise to −COOH bands	Increased	Increased	[61]
1233	j	Epoxy C–O–C	Lowered	Lowered	[58]
1107	k	Not determined or Si–O–Si asymmetric	Appeared	Appeared	[63]
1064	l	–C–OH stretching	Significantly lowered	Significantly lowered	[57]
986	m	Epoxy stretching	Lowered	Significantly lowered	[57]
929	n	Not appeared	Did not appear	C–O trans telescopic vibration	[64,65]

**Table 2:** Effective kinetic constants for the proposed model system. Initial conditions: luminol, 2 mM; H_2_O_2_, 200 μM; GO, 5 μg/L.

Reactive Species		
	NO	NO_2_^−^
Initial concentrations, μM	30	100
**Value of simulated constant, nM^−1^s^−1^**		
Model CL system with nitric oxide (NO) + H_2_O_2_ + Luminol		
1. NO + H_2_O_2_ → ONOO^−^	0.55	Formation of reactive nitrogen species
2. NO_2_^−^ + H_2_O_2_ → ONOO^−^	0.083	
3. NO + H_2_O_2_ → NO_2_^−^ + H_2_O	0.28	NO oxidation
(4a) ONOO^−^ + Lum → Lum*	0.55	Formation of an excited product
(4b) Lum* → P + *hν*	0.28	Luminescence
CL system with GO + nitric oxide + H_2_O_2_ + luminol		
GO + NO → P	1.94	Antioxidant activity
GO + Lum* → P	0.028	CL quenching

## Data Availability

Data are contained within the article.

## Abbreviations

The following abbreviations are used in this manuscript:

(bulk) GO	Purified aqGO using dialysis bag (membrane) 0.5 kDa
(nano) GO	Molecular mass-dependent fractions of aqGO prepared using dialysis bag (membrane) separation in the range of MWCO from 14 to 0.5 kDa
aqGO	Aqueous graphene oxide dispersions
CL	Chemiluminescence
c₁/₂	Concentration showing half-suppression of signal
GO	Graphene oxide
Hb	Hemoglobin
LOQ	Limit of quantification
MetHb	Methemoglobin
MWCO	Molecular-weight cut-off
PBS	Phosphate-buffered saline
SG	Savitzky–Golay
STP	Standard temperature and pressure

## Appendix A

Let us assume the expected density of the graphene oxide as 1.36 $g/cm^{3}$ [133]. The molar mass (M) for the nanographene fraction equals 1 kDa = 1000 g/mol. N (quantity of atoms) is about 1000 divided by 12 is the molar mass of carbon (C) yielding approximately 83.3, which can be rounded up to the nearest 100 atoms. Consider the formula of material density and basic chemical calculus: $d(g/cm^{3})=\frac{m(g)}{V(cm^{3})}$. Avogadro’s number (*N*_A_) is 6.022 × 10^23^ mol^−1^. We can conclude that $d(g/cm^{3})=M\frac{N}{N_{A}}/\frac{4}{3}\pi r^{3}$ and *D* (diameter) equals two radii (*r*). Given that 1 cm is equal to 10^7^ nm, to convert the diameter from centimeters to nanometers, we multiply by 100. The final equation is

$$D=2\sqrt[{3}]{\frac{M\frac{N}{N_{A}}}{\frac{4}{3}\pi d}}\times 100=6.2nm.$$
